# Pb_3_Te_2_O_6_Br_2_

**DOI:** 10.1107/S1600536809053604

**Published:** 2010-01-16

**Authors:** Matthias Weil, Berthold Stöger

**Affiliations:** a Institute for Chemical Technologies and Analytics, Division of Structural Chemistry, Vienna University of Technology, Getreidemarkt 9/164-SC, A-1060 Vienna, Austria

## Abstract

Single crystals of the title compound, trilead(II) bis­[tellurate(IV)] dibromide, have been grown under hydro­thermal conditions. The structure is isotypic with that of the chloride analogue, Pb_3_Te_2_O_6_Cl_2_, and consists of three Pb, two Te, two Br and four O atoms in the asymmetric unit. Except for two of the O atoms, all atoms are located on mirror planes. The Pb_3_Te_2_O_6_Br_2_ structure can be described as being built up from _∞_^2^[Pb_3_Te_2_O_6_]^2+^ layers extending parallel to (20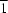) and Br^−^ anions between the layers. Cohesion of the structure is accomplished through Pb—Br contacts of two of the three lead atoms, leading to highly asymmetric coordination polyhedra. The lone-pair electrons of both Te^IV^ and Pb^II^ atoms are stereochemically active and point towards the anionic halide layers.

## Related literature

For reports and structures of other compounds in the system Pb*X*_2_—PbO—TeO_2_, where *X* = Br, Cl, see: Pb_3_TeO_4_ *X*_2_ (Charkin *et al.*, 2006 ▶; Porter & Halasyamani, 2003 ▶); Pb_3_Te_2_O_6_ *X*_2_ (Porter & Halasyamani, 2003 ▶). The crystal chemistry of oxotellurate(IV) compounds has been reviewed by Dolgikh (1991 ▶) and Zemann (1971 ▶). For other oxotellurates(IV) prepared under hydro­thermal conditions, see: Weil & Stöger (2007 ▶, 2008**a* ▶,b* ▶).

## Experimental

### 

#### Crystal data

Pb_3_Te_2_O_6_Br_2_
*M* *_r_* = 1132.59
Monoclinic, 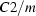

*a* = 16.9151 (9) Å
*b* = 5.6813 (3) Å
*c* = 11.0623 (6) Å
β = 104.046 (1)°
*V* = 1031.3 (1) Å^3^
*Z* = 4
Mo *K*α radiation
μ = 62.14 mm^−1^
*T* = 296 K
0.18 × 0.10 × 0.04 mm

#### Data collection

Bruker APEXII CCD diffractometer
Absorption correction: numerical (*HABITUS*; Herrendorf, 1997 ▶) *T*_min_ = 0.06, *T*_max_ = 0.41
3758 measured reflections
1371 independent reflections
1338 reflections with *I* > 2σ(*I*)
*R*_int_ = 0.030

#### Refinement

*R*[*F* ^2^ > 2σ(*F* ^2^)] = 0.048
*wR*(*F* ^2^) = 0.107
*S* = 1.15
1371 reflections
73 parameters
Δρ_max_ = 7.41 e Å^−3^
Δρ_min_ = −6.41 e Å^−3^

### 

Data collection: *APEX2* (Bruker, 2008 ▶); cell refinement: *SAINT* (Bruker, 2008 ▶); data reduction: *SAINT*; program(s) used to solve structure: *SHELXS97* (Sheldrick, 2008 ▶); program(s) used to refine structure: *SHELXL97* (Sheldrick, 2008 ▶); molecular graphics: *ATOMS* (Dowty, 2006 ▶); software used to prepare material for publication: *SHELXL97*.

## Supplementary Material

Crystal structure: contains datablocks I, global. DOI: 10.1107/S1600536809053604/br2129sup1.cif

Structure factors: contains datablocks I. DOI: 10.1107/S1600536809053604/br2129Isup2.hkl

Additional supplementary materials:  crystallographic information; 3D view; checkCIF report

## Figures and Tables

**Table 1:** Comparative geometrical parameters (Å) for selected bond lengths in Pb_3_Te_2_O_6_ *X*_2_ compounds (*X* = Br, Cl)

Distance	*X*= Br (this work)	*X*= Cl (Porter & Halasyamani, 2003 ▶)
Pb1—O4	2.415 (11)	2.447 (15)
Pb1—O1	2.617 (14)	2.586 (14)
Pb1—*X*2^i^	3.274 (3)	3.176 (12)
Pb1—*X*1	3.3287 (13)	3.237 (12)
Pb1—*X*1^ii^	3.364 (3)	3.247 (12)
Pb2—O1	2.360 (13)	2.374 (14)
Pb2—O3^iii^	2.451 (18)	2.48 (2)
Pb2—O2	2.704 (19)	2.677 (13)
Pb2—*X*2	3.3955 (17)	3.270 (12)
Pb2—*X*1^iv^	3.415 (3)	3.276 (13)
Pb3—O4	2.555 (12)	2.544 (16)
Pb3—O1	2.617 (13)	2.600 (14)
Pb3—O4^v^	2.788 (11)	2.777 (17)
Pb3—O3^iii^	2.995 (8)	2.986 (16)
Pb3—*X*2	3.338 (21)	3.244 (12)
Te1—O1	1.989 (13)	1.938 (13)
Te1—O2	2.085 (17)	2.044 (12)
Te2—O3	1.874 (19)	1.84 (2)
Te2—O4	1.879 (11)	1.861 (16)

Symmetry codes: (i) *x* + 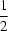, *y* − 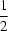, *z*; (ii) −*x* + 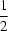, −*y* + 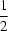, −*z*; (iii) −*x* + 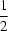, −*y* + 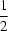, −*z* + 1; (iv) *x* − 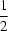, *y* − 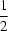, *z*; (v) −*x* + 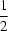, *y* + 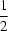, −*z* + 1.

**Fig. 1.**
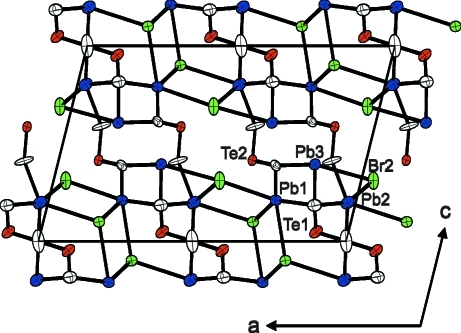
The crystal structure of Pb3Te2O6Br2 in projection along [010]. Te atoms are given as red, Pb as blue, O atoms as white and Br atoms as green ellipsoids at the 74% probability level.

## supplementary crystallographic information

### Comment

Single crystals growth of Pb_3_Te_2_O_6_Cl_2_ and Pb_3_Te_2_O_6_Br_2_ was studied during a recent project intended to elaborate hydrothermal formation conditions of oxotelluraTe^IV^ compounds (Weil & Stöger, 2007; 2008*a,b*). Both Pb_3_Te_2_O_6_*X*_2_ (*X* = Br, Cl) compounds have been prepared previously by solid state techniques (Porter & Halasyamani, 2003). Whereas for the chloride compound a full structure analysis was undertaken at that time, for the isotypic bromide compound only lattice parameters were reported. Here we present details of the Pb_3_Te_2_O_6_Br_2_ structure determined from single-crystal X-ray data.

The asymmetric unit of the Pb_3_Te_2_O_6_*X*_2_ structure contains three Pb, two Te, two *X* and four O atoms. Except two of the O atoms, all other atoms are located on mirror planes. The Pb_3_Te_2_O_6_*X*_2_ structure type can be described as being built up from ^2^_∞_[Pb_3_Te_2_O_6_]^2+^ layers extending parallel to (201) and *X*^-^ anions between the layers. The tellurium atoms in the cationic layer are surrounded by four (Te1) and three (Te2) oxygen atoms in distorted tetrahedral (Te1) and trigonal-pyramidal (Te2) environments, respectively. Under an additional contribution of the lone electron pairs to the stereochemistry of the two Te^IV^ atoms, the corresponding Ψ-oxopolyhedra can be considered as distorted TeO_4_*E* square pyramids (Te1) and TeO_3_*E* tetrahedra (*E* designates the lone electron pair). These kinds of TeO*_x_* polyhedra are frequently observed for various oxotelluraTe^IV^ structures (Dolgikh, 1991; Zemann, 1971). The Te1O_4_ group forms Te1_2_O_6_ dimers *via* edge-sharing, whereas the Te2O_3_ group is isolated in the layers. Both oxotelluraTe^IV^ units are surrounded by lead atoms. Four O atoms are bonded to Pb1, five O atoms to Pb2 and eight O atoms to Pb3. For both Pb_3_Te_2_O_6_*X*_2_ structures the respective Te—O and Pb—O distances are very similar (Table 1). The main difference between the Pb_3_Te_2_O_6_*X*_2_ structures pertains to the distances of the lead atoms to the *X* atoms that are situated between the cationic layers. As expected, the Pb—*X* distances are about 0.1 to 0.15 Å longer for the Br compound (Table 1). The layered character of the Pb_3_Te_2_O_6_*X*_2_ structure type with alternating layers parallel to (201) is also reflected by the differences of the lattice parameters for the Br and the Cl analogues. While the lengths of the *b*-axes are very similar (5.6813 (3) (Br) *versus* 5.6295 (4) Å (Cl)), *a* and *c* differ notedly (16.9151 (9) *versus* 16.4417 (11) Å, and 11.0623 (6) *versus* 10.8894 (7) Å) due to the different ionic radii of Br^-^ and Cl^-^. The coordination of the halogen atoms in the neighbouring anionic layer to the lead atoms augments the coordination polyhedra of Pb1 and Pb2 to an overall coordination of [Pb1O_4_*X*_4_], [Pb2O_5_*X*_3_] and [Pb3O_8_*X*].

All TeO*_x_* and PbO*_x_X_y_* polyhedra in the structure are highly irregular. The lone-pair electrons of both Te^IV^ and Pb^II^ atoms are stereochemically active and point towards the anionic halide layers (Fig. 1). Similar TeO*_x_* and PbO*_x_X_y_* polyhedra are observed for the Pb_3_TeO_4_*X*_2_ structures (Charkin *et al.*, 2006) that show a lower TeO_2_ content in comparison with the Pb_3_Te_2_O_6_*X*_2_ structures.

### Experimental

All chemicals used were of analytical grade and employed without further purification. 1 mmol PbBr_2_ (0.367 g) and 1 mmol TeO_2_ (0.160 g) were placed in a Teflon inlay that was filled with a hydrous NH_4_OH solution (10%_wt_) to two-thirds of its volume. The inlay was placed in a steel autoclave and heated at 493 K for four weeks. The reaction product consisted of small colourless crystals of the title compound with rod-like habit and a maximum edge lengths of 0.2 mm. Experiments under similar conditions but with with PbCl_2_ instead of PbBr_2_ led to crystals of the isotypic compound Pb_3_Te_2_O_6_Cl_2_.

### Refinement

The structure was solved using direct methods. For better comparison with the isotypic structure of Pb_3_Te_2_O_6_Cl_2_ (Porter & Halasyamani, 2003), the atomic coordinates of the Cl analogue were taken as starting parameters for refinement. The highest remaining peak in the final difference Fourier map is 0.80 Å from Pb2 and the deepest hole is 0.57 Å from the same atom.

Lattice parameters of Pb_3_Te_2_O_6_Br_2_ based on the present single-crystal study agree reasonably well with those of the powder diffraction data provided by Porter & Halasyamani (2003): *a* = 16.8911 (8), *b* = 5.6804 (2), *c* = 11.0418 (5) Å, *β* = 104.253 (2)°.

### Crystal data

**Table:**

Pb_3_Te_2_O_6_Br_2_	*F*(000) = 1872
*M**_r_* = 1132.59	*D*_x_ = 7.295 Mg m^−^^3^
Monoclinic, *C*2/*m*	Mo *K*α radiation, λ = 0.71073 Å
Hall symbol: -C 2y	Cell parameters from 4771 reflections
*a* = 16.9151 (9) Å	θ = 2.5–32.2°
*b* = 5.6813 (3) Å	µ = 62.14 mm^−^^1^
*c* = 11.0623 (6) Å	*T* = 296 K
β = 104.046 (1)°	Rod, colourless
*V* = 1031.3 (1) Å^3^	0.18 × 0.10 × 0.04 mm
*Z* = 4	

### Data collection

**Table:**

Bruker APEXII CCD diffractometer	1371 independent reflections
Radiation source: fine-focus sealed tube	1338 reflections with *I* > 2σ(*I*)
graphite	*R*_int_ = 0.030
ω– and φ–scans	θ_max_ = 27.9°, θ_min_ = 1.9°
Absorption correction: numerical (*HABITUS*; Herrendorf, 1997)	*h* = −22→15
*T*_min_ = 0.06, *T*_max_ = 0.41	*k* = −7→7
3758 measured reflections	*l* = −13→14

### Refinement

**Table:**

Refinement on *F*^2^	Primary atom site location: structure-invariant direct methods
Least-squares matrix: full	Secondary atom site location: difference Fourier map
*R*[*F*^2^ > 2σ(*F*^2^)] = 0.048	*w* = 1/[σ^2^(*F*_o_^2^) + (0.0166*P*)^2^ + 250.8836*P*] where *P* = (*F*_o_^2^ + 2*F*_c_^2^)/3
*wR*(*F*^2^) = 0.107	(Δ/σ)_max_ < 0.001
*S* = 1.15	Δρ_max_ = 7.41 e Å^−^^3^
1371 reflections	Δρ_min_ = −6.41 e Å^−^^3^
73 parameters	Extinction correction: *SHELXL97* (Sheldrick, 2008), Fc^*^=kFc[1+0.001xFc^2^λ^3^/sin(2θ)]^-1/4^
0 restraints	Extinction coefficient: 0.00021 (2)

### Special details

**Table:**

Geometry. All e.s.d.'s (except the e.s.d. in the dihedral angle between two l.s. planes) are estimated using the full covariance matrix. The cell e.s.d.'s are taken into account individually in the estimation of e.s.d.'s in distances, angles and torsion angles; correlations between e.s.d.'s in cell parameters are only used when they are defined by crystal symmetry. An approximate (isotropic) treatment of cell e.s.d.'s is used for estimating e.s.d.'s involving l.s. planes.
Refinement. Refinement of *F*^2^ against ALL reflections. The weighted *R*-factor *wR* and goodness of fit *S* are based on *F*^2^, conventional *R*-factors *R* are based on *F*, with *F* set to zero for negative *F*^2^. The threshold expression of *F*^2^ > σ(*F*^2^) is used only for calculating *R*-factors(gt) *etc*. and is not relevant to the choice of reflections for refinement. *R*-factors based on *F*^2^ are statistically about twice as large as those based on *F*, and *R*- factors based on ALL data will be even larger.

### Fractional atomic coordinates and isotropic or equivalent isotropic displacement parameters (Å^2^)

**Table:**

	*x*	*y*	*z*	*U*_iso_*/*U*_eq_	
Pb1	0.26158 (6)	0.0000	0.21028 (8)	0.0178 (2)	
Pb2	0.02616 (6)	0.0000	0.19788 (9)	0.0250 (3)	
Pb3	0.16368 (6)	0.5000	0.39320 (8)	0.0217 (3)	
Te1	0.10510 (10)	0.5000	0.04764 (16)	0.0233 (4)	
Te2	0.37019 (9)	0.5000	0.41467 (14)	0.0141 (3)	
Br1	0.31858 (15)	0.5000	0.0993 (2)	0.0205 (5)	
Br2	−0.03969 (16)	0.5000	0.3103 (3)	0.0313 (6)	
O1	0.1315 (8)	0.263 (3)	0.1840 (11)	0.028 (3)	
O2	0.0000	0.294 (5)	0.0000	0.059 (7)	
O3	0.3884 (15)	0.5000	0.5887 (17)	0.040 (5)	
O4	0.2909 (7)	0.262 (2)	0.3877 (10)	0.017 (2)	

### Atomic displacement parameters (Å^2^)

**Table:**

	*U*^11^	*U*^22^	*U*^33^	*U*^12^	*U*^13^	*U*^23^
Pb1	0.0224 (5)	0.0150 (4)	0.0161 (4)	0.000	0.0048 (3)	0.000
Pb2	0.0258 (5)	0.0204 (5)	0.0253 (5)	0.000	−0.0004 (4)	0.000
Pb3	0.0190 (4)	0.0261 (5)	0.0191 (4)	0.000	0.0026 (3)	0.000
Te1	0.0224 (8)	0.0186 (8)	0.0236 (8)	0.000	−0.0046 (6)	0.000
Te2	0.0117 (6)	0.0124 (7)	0.0177 (7)	0.000	0.0025 (5)	0.000
Br1	0.0208 (11)	0.0220 (11)	0.0183 (11)	0.000	0.0039 (9)	0.000
Br2	0.0202 (12)	0.0236 (13)	0.0525 (18)	0.000	0.0131 (12)	0.000
O1	0.032 (7)	0.026 (7)	0.023 (6)	−0.010 (6)	0.005 (5)	0.002 (5)
O2	0.031 (11)	0.062 (18)	0.09 (2)	0.000	0.028 (13)	0.000
O3	0.051 (13)	0.046 (14)	0.011 (8)	0.000	−0.014 (8)	0.000
O4	0.019 (5)	0.016 (6)	0.018 (5)	−0.007 (5)	0.009 (4)	−0.001 (4)

### Geometric parameters (Å, °)

**Table:**

Pb1—O4	2.415 (11)	Pb3—O4^viii^	2.555 (12)
Pb1—O4^i^	2.415 (11)	Pb3—O4	2.555 (12)
Pb1—O1^i^	2.617 (14)	Pb3—O1	2.617 (13)
Pb1—O1	2.617 (14)	Pb3—O1^viii^	2.617 (13)
Pb1—Br2^ii^	3.274 (3)	Pb3—O4^ix^	2.788 (11)
Pb1—Br1^iii^	3.3287 (13)	Pb3—O4^v^	2.788 (11)
Pb1—Br1	3.3287 (13)	Pb3—O3^v^	2.995 (8)
Pb1—Br1^iv^	3.364 (3)	Pb3—O3^x^	2.995 (8)
Pb2—O1	2.360 (13)	Pb3—Te2	3.4432 (17)
Pb2—O1^i^	2.360 (13)	Te1—O1^viii^	1.989 (13)
Pb2—O3^v^	2.451 (18)	Te1—O1	1.989 (13)
Pb2—O2^vi^	2.704 (19)	Te1—O2	2.085 (17)
Pb2—O2	2.704 (19)	Te1—O2^xi^	2.085 (17)
Pb2—Br2	3.3955 (17)	Te2—O3	1.874 (19)
Pb2—Br2^iii^	3.3955 (17)	Te2—O4	1.879 (11)
Pb2—Br1^vii^	3.415 (3)	Te2—O4^viii^	1.879 (11)
			
O4—Pb1—O4^i^	76.0 (5)	O1—Te1—O2	80.4 (6)
O4—Pb1—O1^i^	116.3 (4)	O1^viii^—Te1—O2^xi^	80.4 (6)
O4^i^—Pb1—O1^i^	74.9 (4)	O1—Te1—O2^xi^	126.3 (5)
O4—Pb1—O1	74.9 (4)	O2—Te1—O2^xi^	68.2 (14)
O4^i^—Pb1—O1	116.3 (4)	O3—Te2—O4	95.5 (6)
O1^i^—Pb1—O1	69.7 (6)	O3—Te2—O4^viii^	95.5 (6)
O1—Pb2—O1^i^	78.6 (7)	O4—Te2—O4^viii^	92.3 (7)
O1—Pb2—O3^v^	77.6 (5)	Te1—O1—Pb2	116.3 (6)
O1^i^—Pb2—O3^v^	77.6 (5)	Te1—O1—Pb1	119.9 (6)
O1—Pb2—O2^vi^	108.5 (4)	Pb2—O1—Pb1	105.0 (5)
O1^i^—Pb2—O2^vi^	62.2 (4)	Te1—O1—Pb3	106.4 (6)
O3^v^—Pb2—O2^vi^	136.2 (5)	Pb2—O1—Pb3	105.4 (5)
O1—Pb2—O2	62.2 (4)	Pb1—O1—Pb3	101.9 (4)
O1^i^—Pb2—O2	108.5 (4)	Te1—O2—Te1^xi^	111.8 (14)
O3^v^—Pb2—O2	136.2 (5)	Te1—O2—Pb2^vi^	120.87 (15)
O2^vi^—Pb2—O2	76.4 (10)	Te1^xi^—O2—Pb2^vi^	100.36 (16)
O4^viii^—Pb3—O4	64.0 (5)	Te1—O2—Pb2	100.36 (16)
O4^viii^—Pb3—O1	104.3 (4)	Te1^xi^—O2—Pb2	120.87 (15)
O4—Pb3—O1	72.7 (4)	Pb2^vi^—O2—Pb2	103.6 (10)
O4^viii^—Pb3—O1^viii^	72.7 (4)	Te2—O3—Pb2^v^	154.3 (13)
O4—Pb3—O1^viii^	104.3 (4)	Te2—O4—Pb1	124.8 (5)
O1—Pb3—O1^viii^	61.9 (6)	Te2—O4—Pb3	100.8 (5)
O1^viii^—Te1—O1	85.1 (8)	Pb1—O4—Pb3	109.7 (4)
O1^viii^—Te1—O2	126.3 (5)		

Symmetry codes: (i) *x*, −*y*, *z*; (ii) *x*+1/2, *y*−1/2, *z*; (iii) *x*, *y*−1, *z*; (iv) −*x*+1/2, −*y*+1/2, −*z*; (v) −*x*+1/2, −*y*+1/2, −*z*+1; (vi) −*x*, −*y*, −*z*; (vii) *x*−1/2, *y*−1/2, *z*; (viii) *x*, −*y*+1, *z*; (ix) −*x*+1/2, *y*+1/2, −*z*+1; (x) −*x*+1/2, −*y*+3/2, −*z*+1; (xi) −*x*, −*y*+1, −*z*.

### Table 1 Comparative geometrical parameters (Å) for selected bond lengths in Pb_3_Te_2_O_6_*X*_2_ compounds (*X* = Br, Cl)

**Table:**

Distance	*X*= Br (this work)	*X*= Cl (Porter & Halasyamani, 2003)
Pb1—O4	2.415 (11)	2.447 (15)
Pb1—O1	2.617 (14)	2.586 (14)
Pb1—*X*2^i^	3.274 (3)	3.176 (12)
Pb1—*X*1	3.3287 (13)	3.237 (12)
Pb1—*X*1^ii^	3.364 (3)	3.247 (12)
Pb2—O1	2.360 (13)	2.374 (14)
Pb2—O3^iii^	2.451 (18)	2.48 (2)
Pb2—O2	2.704 (19)	2.677 (13)
Pb2—*X*2	3.3955 (17)	3.270 (12)
Pb2—*X*1^iv^	3.415 (3)	3.276 (13)
Pb3—O4	2.555 (12)	2.544 (16)
Pb3—O1	2.617 (13)	2.600 (14)
Pb3—O4^v^	2.788 (11)	2.777 (17)
Pb3—O3^iii^	2.995 (8)	2.986 (16)
Pb3—*X*2	3.338 (21)	3.244 (12)
Te1—O1	1.989 (13)	1.938 (13)
Te1—O2	2.085 (17)	2.044 (12)
Te2—O3	1.874 (19)	1.84 (2)
Te2—O4	1.879 (11)	1.861 (16)

Symmetry codes: (i) x+1/2, y-1/2, z; (ii) -x+1/2, -y+1/2, -z; (iii) -x+1/2, -y+1/2, -z+1; (iv) x-1/2, y-1/2, z; (v) -x+1/2, y+1/2, -z+1.
